# New material of named fossil turtles from the Late Jurassic (late Kimmeridgian) of Wattendorf, Germany

**DOI:** 10.1371/journal.pone.0233483

**Published:** 2020-06-03

**Authors:** Walter G. Joyce, Matthias Mäuser

**Affiliations:** 1 Departement für Geowissenschaften, Universität Freiburg, Freiburg, Switzerland; 2 Naturkunde-Museum Bamberg, Staatliche Naturwissenschaftliche Sammlungen Bayerns, Bamberg, Germany; Indiana University Bloomington, UNITED STATES

## Abstract

The newly discovered plattenkalk (platy limestone) locality of Wattendorf, southern Germany, has yielded a diverse fauna and flora dated to the base of the late Kimmeridgian, Late Jurassic. We here describe three fossil turtle specimens that were recovered during systematic excavations of a distinct, 15 cm thick package of plattenkalks by the Naturkunde-Museum Bamberg. The first specimen is a large shell of *Achelonia formosa*, a taxon that is based on material from the late Kimmeridgian of Cerin, France. The new specimen suggests synonymy with *Enaliochelys chelonia* from the late Kimmeridgian of the United Kingdom. The second is a near-complete skeleton of the enigmatic *Tropidemys seebachi*, which was previously known only from the late Kimmeridgian of Hannover, northern Germany. The third specimen is a partial skeleton of *Eurysternum wagleri*, which had previously been known only from the early Tithonian of the Solnhofen region, southern Germany. In addition to new anatomical insights, the new material provides further evidence for spatial links during the late Kimmeridgian between northern and southern Germany, France, and the United Kingdom and temporal link from the late Kimmeridgian to the early Tithonian. The prevalence of partial, though articulated specimens is suggestive of predation by an unknown large marine reptile.

## Introduction

Late Jurassic deposits across Europe have yielded an astounding array of fossil turtles since the 19th century (see [1, 2] for recent summaries) that were recently united into the clade Thalassochelydia [1]. Several factors have hindered a meaningful synthesis of this fauna. First, fossil turtle taxa were often named over the course of the 19th century based on highly fragmentary material [3, 4, 5], but generations of workers have been reluctant to ignore the resulting dubious names [6, 7] resulting in a pantheon of poorly diagnosed taxa [1]. Second, many Late Jurassic turtles have been recovered as partial to complete skeletons from platy limestones, but the associated cranial material is typically crushed and specimens only prepared from one side (e.g., [8–14]), making comparison difficult with three-dimensionally preserved specimens from other localities and specimens preserved in opposite views (dorsal versus ventral). Third, significant collections of Late Jurassic turtles were mentioned and named in the literature (e.g., [15]), but never figured, making it impossible for the greater community to evaluate their morphology. Finally, although some comparisons were made across borders, there was a certain tendency over the course of the 19th century to recognize local faunas defined by national boundaries [1]. A revived interest in the turtles of the Late Jurassic of Europe has recently been able to clarify the morphology of much historic material [16–23] and to establish a network of occurrences across Europe [21, 22, 24, 25], but the morphology, status, and distribution of many taxa remains unresolved.

**Fig 1:**
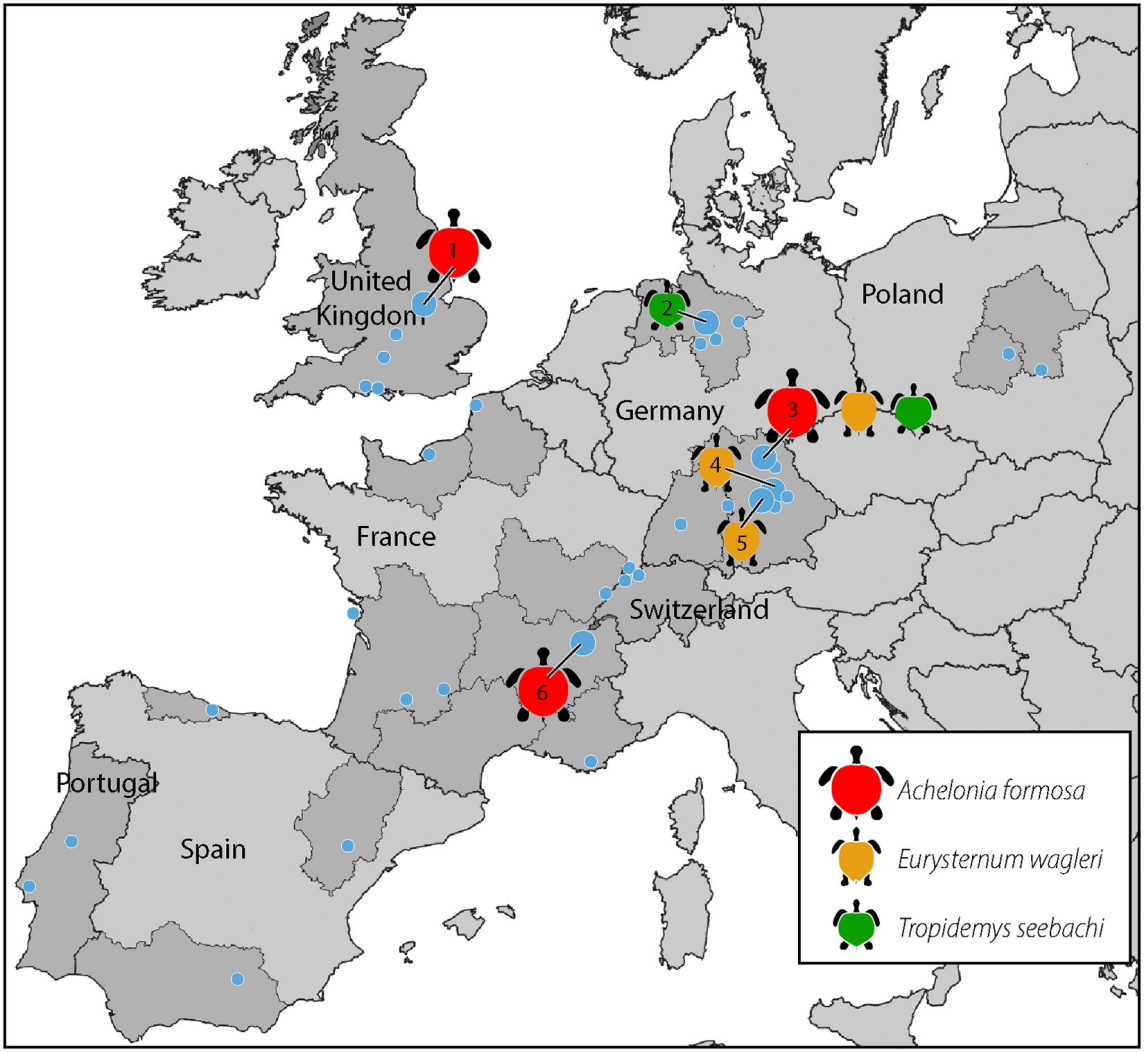
Simplified map of the distribution of the fossil turtles *Achelonia formosa*, *Eurysternum wagleri*, and *Tropidemys seebachi*. All points signify previously documented fossil turtle localities following Anquetin et al. (2017). The map was created using Maperitive. Larger dots represent localities explicitly discussed in the text: 1, Ely, United Kingdom; 2, Hannover, Germany; 3, Wattendorf, Germany; 4, Denkendorf/Zandt, Germany; 5, Solnhofen, Germany; 6, Cerin, France.

The plattenkalks (i.e., platy limestones) of Central Europe have been one of the primary sources of thalassochelydians, the most famous of which are those of the Southern Franconian Alb around the village of Solnhofen and the town of Eichstätt, Germany. Although there is a tendency to view the fauna of the plattenkalks as a single occurrence, more recent sedimentological and stratigraphic work has concluded that these localities form a band of spatially disjunct micro-basins that range stratigraphically from the Late Kimmeridgian to the early Tithonian and geographically from Canjuers in southeastern France, to Cerin in east-central France, Nusplingen in southwestern Germany, and the localities of Eichstätt, Kehlheim, Schamhaupten, Solnhofen, Zandt, Painten, and Brunn, among others, in southeastern Germany (Fig 1). The recently discovered locality of Wattendorf in northern Bavaria significantly expands this range by being situated about 120 km north of the nearest localities in central Bavaria and by being dated to the base of the Late Kimmeridgian [26, 27]. Systematic excavations at this site, conducted by the Naturkunde-Museum Bamberg since the year 2004, have yielded an exceptional fauna that to date includes 16 turtles representing at least eight species. The purpose of this contribution is to describe the three turtle remains from this locality that represent previously named taxa and to discuss the taxonomic and biogeographic implications of these specimens. Two of these specimens had previously been noted in the literature [28–30], but their comprehensive illustration, description, or discussion is still outstanding.

### Institutional abbreviations

BSPG, Bayerische Staatssammlung für Paläontologie und Geologie, Munich, Germany; CM, Carnegie Museum, Pittsburg, Pennsylvania, USA; CAMSM, Sedgwick Museum, Cambridge, United Kingdom; GZG, Geowissenschaftliches Zentrum Göttingen, Göttingen, Germany; MHNL, Museum d’Histoire Naturelle de Lyon, Lyon, France; NKMB, Naturkunde-Museum Bamberg, Bamberg, Germany.

## Geological settings

The locality of Wattendorf is located in the limestone/dolomite quarry of the Andreas Schorr GmbH & Co. KG just outside of the village of Wattendorf, about 25 km northeast of the city of Bamberg in Bavaria, Germany (Fig 1). The quarry, which covers an area of about 30 hectares, exposes massive dolostones in its northeastern area and bedded limestones in its southwestern part. Similar to other localities in the region, the dolostones are composed of microbialite-sponge reefs. The dolostones dip towards the east and form the base of an internal reef basin, the so-called Wattendorf Basin (*Wattendorfer Wanne* of [29]). This basin is filled mostly by the bedded Wattendorf limestones (*Wattendorfer Kalk*, sensu [31]), which belongs to the Torleite Formation [32, 33]). A sequence of plattenkalks, which alternate with layers of coarse-grained reef debris, turbidites and dolobindstones, is sandwiched between the dolostones below and at least 30 m of bedded limestone above. At the excavation site, this sequence has a thickness of about 6 m, but thins out towards the microbialithe-sponge reefs in the northeastern part of the quarry and turns to zero at the top of the adjoining reefs.

Most of the fossils recovered during systematic excavations of the Naturkunde-Museum Bamberg, also the herein described turtles, originate from a distinctive package of plattenkalks of 15 cm thickness, which is located about 1 m under the base of the Wattendorf limestone. These plattenkalks consist of fine-grained beds, microbial laminae, and thin clay laminae. There is no bioturbation. Terrestrial plants are common and suggest general proximity to land, either small islands or the nearby Bohemian or Central German landmasses. Invertebrates mostly include brachiopods, cephalopods, bivalves, gastropods, and echinoids. In addition to turtles, the vertebrate fauna consists of a broad diversity of fish, but also rare pterosaurs, crocodilians, and sphenodontians [29].

The lack of bioturbation and ichnofauna in association with the perfect conservation of articulated vertebrates suggest that conditions were hostile to life at the seafloor at the time of sedimentation. All organisms therefore descended from the water column above where normal conditions prevailed [29].

## Legal status

The Naturkunde-Museum Bamberg (NKMB) has been conducting annual excavations at Wattendorf since 2004 with explicit permission from Andreas Schorr GmbH & Co. KG which have been regulated by a series of contracts. The vast majority of fossil finds, in particular all invertebrates and most fish, legally belong to NKMB, but ownership of all specimens with greater monetary value is regulated on an individual basis. The three turtles presented herein are the legal property of the company but are on permanent loan to NKMB for study. In the case of an intended sale by the company, the museum holds an explicit option right, which it is highly likely to take, as the excavation at Wattendorf enjoys broad support from the state and the public, also in the form of donations. Although the final destination of these finds has yet to be determined, five possibilities remain the most likely: 1) the status quo is retained; 2) the fossils are donated to NKMB; 3) the fossils are sold to NKMB or another public repository; 4) the fossils remain in private hand, but are declared a cultural treasure (*Kulturgut*) following Bavarian or German law, which guarantees access to scientists; 5) the fossils are sold to a private owner, who may or may not provide access to the public. Either of the first four options is the most plausible outcome and access to these specimens therefore seems all but guaranteed for the future. It is only because we have such high confidence in the continued access that we present the material to the scientific public. However, in the unlikely event that the specimens are forced to leave the public sphere, NKMB will retain archival casts associated with high-quality digital documentation, which will be made available in a public repository.

## Systematic paleontology

TESTUDINATA Klein, 1760 [34]

THALASSOCHELYDIA Anquetin et al., 2017 [1]

*Achelonia* Meyer, 1860 [11]

**Type species.** *Achelonia formosa* Meyer, 1860 [11].

*Achelonia formosa* Meyer, 1860 [11] (= *Enaliochelys chelonia* Seeley, 1869 [15])

Fig 2

**Fig 2:**
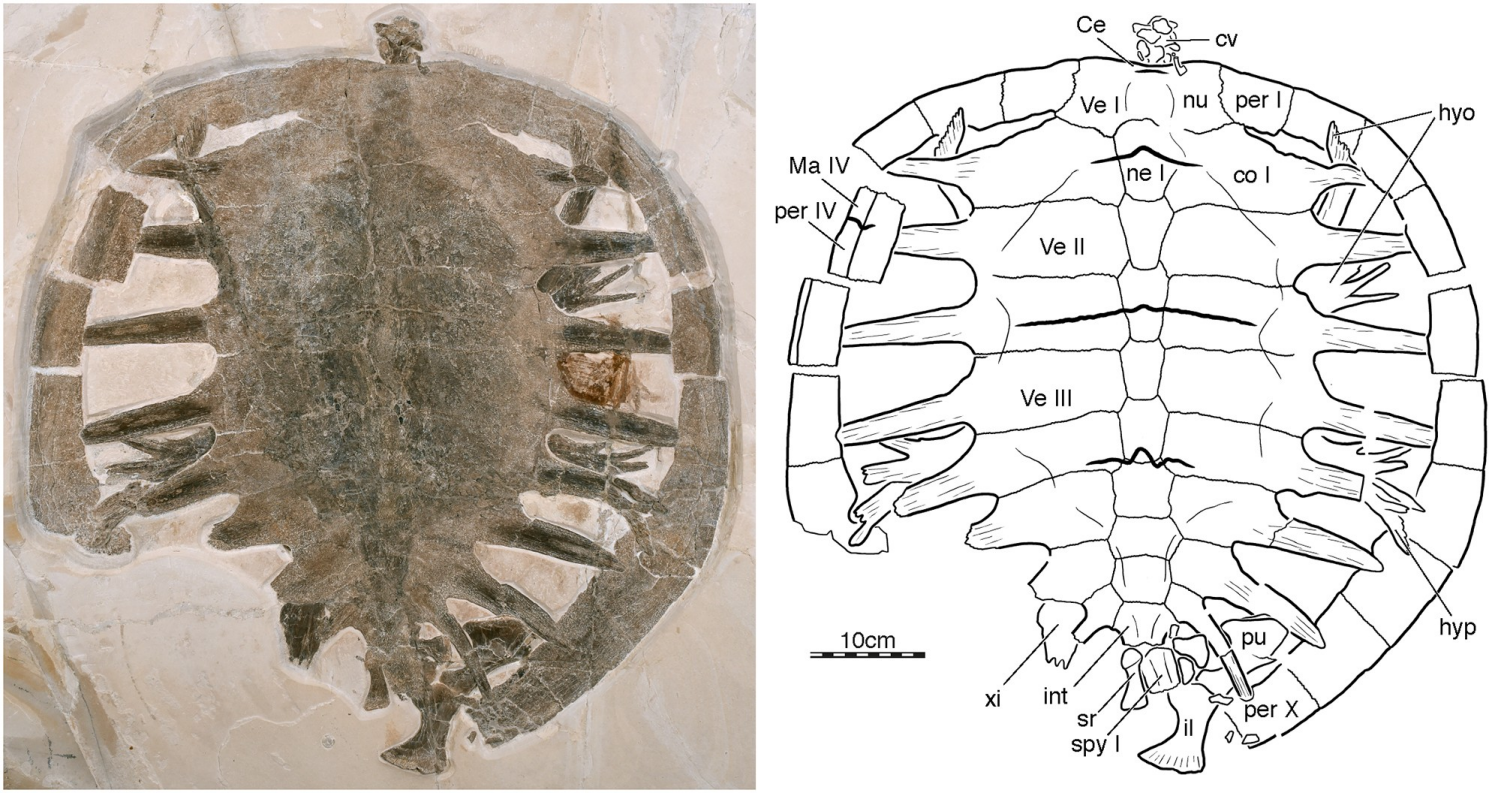
NKMB Watt15/1, *Achelonia formosa*, late Kimmeridgian of Wattendorf, Germany. Photograph and interpretive line drawing in dorsal view. Abbreviations: Ce, cervical scute; co, costal; cv, cervical vertebra; hyo, hyoplastron; hyp, hypoplastron; il, ilium; int, intermediate element; Ma, marginal scute; ne, neural; nu, nuchal; per, peripheral; pu, pubis; spy, suprapygal; sr, sacral rib; Ve, vertebral scute; xi, xiphiplastron.

**Type specimen.** MHNL 20015606 (lectotype), a partial skeleton consisting of the skull, the left forelimb, and the left anterior portions of the shell [11, pl. 17.5], designated by [1].

**Type locality and horizon.** Cerin, Department of Ain, France [11]; Cerin lithographic limestones, late Kimmeridgian, Late Jurassic [35].

**Distribution.** Late Jurassic (late Kimmeridgian) of Cerin, France (lectotype), Ely, Cambridgeshire, United Kingdom (holotype of *Enaliochelys chelonia* Seeley, 1869 [15, 21], and Wattendorf, Bavaria, Germany (specimen referred herein).

**Diagnosis.** *Achelonia formosa* can differentiated from all other known representatives of Thalassochelydia by the following combination of characteristics: large size (i.e., a carapace length of more than 60cm), poorly imprinted carapacial scutes, eight pairs of elongated and flattened costal rib ends associated with nine pairs of carapacial fontanelles, and a ligamentous bridge with lateral plastral fontanelles.

**Comments.** See Description below for the morphology of the new specimens from Wattendorf and the Discussion regarding the proposed synonymy of *Enaliochelys chelonia* Seeley, 1869 [15] with *Achelonia formosa* Meyer, 1860 [11].

*Eurysternum* Meyer, 1839 [8]

**Type species.** *Eurysternum wagleri* Meyer, 1839 [8]

*Eurysternum wagleri* Meyer, 1839 [8] (= *Acichelys redenbacheri* Meyer, 1854 [10])

Fig 3

**Fig 3:**
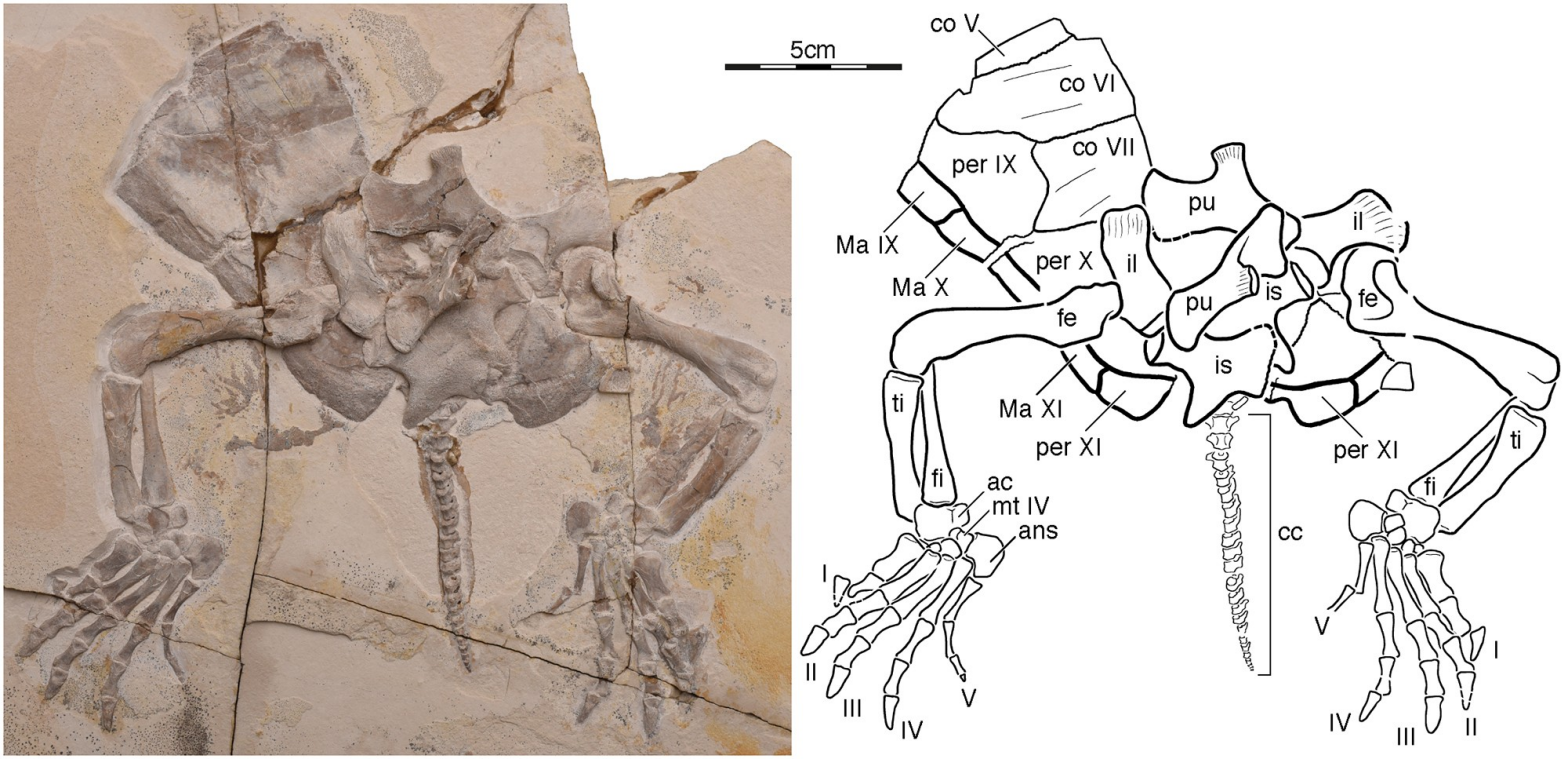
NKMB Watt08/406, *Eurysternum wagleri*, late Kimmeridgian of Wattendorf, Germany. Photograph and interpretive line drawing of specimen in ventral view. Abbreviations: ac, astragalocalcaneum; ans, ansula; cc, caudal column; co, costal; fe, femur; fi, fibula; il, ilium; is, ischium; Ma, marginal scute; mt, metatarsal; per, peripheral; pu, pubis; ti, tibia. Roman numerals label digits.

**Type specimen.** BSPG uncat. (holotype), a disarticulated, partial skeleton [8], now lost [18].

**Type locality and horizon.** Solnhofen, Bavaria, Germany [8]; Altmühltal Formation (a.k.a. Solnhofen Formation), early Tithonian (*Hybonotum* zone), Late Jurassic [36].

**Distribution.** Late Jurassic (early Tithonian) of Solnhofen (holotype) and Denkendorf/Zandt (referred material of [18]), and Late Jurassic (late Kimmeridgian) of Wattendorf, Bavaria, Germany (specimen referred herein).

**Diagnosis.** *Eurysternum wagleri* can most easily be differentiated from all other known representatives of Thalassochelydia by the following combination of characteristics: a thin, pentagonal shell of intermediate size (carapace length up to 50cm), a deep pygal notch, reduced carapacial fontanelles in adults, contribution of vertebral V to posterior margin of shell, a ligamentous bridge, well developed plastral fontanelles, and poor connection of the epi/entoplastron with the hyoplastra (for a more extensive diagnosis see [1]).

**Comments.** See Description below for the morphology of the new specimens from Wattendorf and Discussion for the extended stratigraphic range of the species.

*Tropidemys* Rütimeyer, 1873 [37]

**Type species.** *Tropidemys langii* Rütimeyer, 1873 [37].

*Tropidemys seebachi* Portis, 1878 [5]

Fig 4

**Fig 4:**
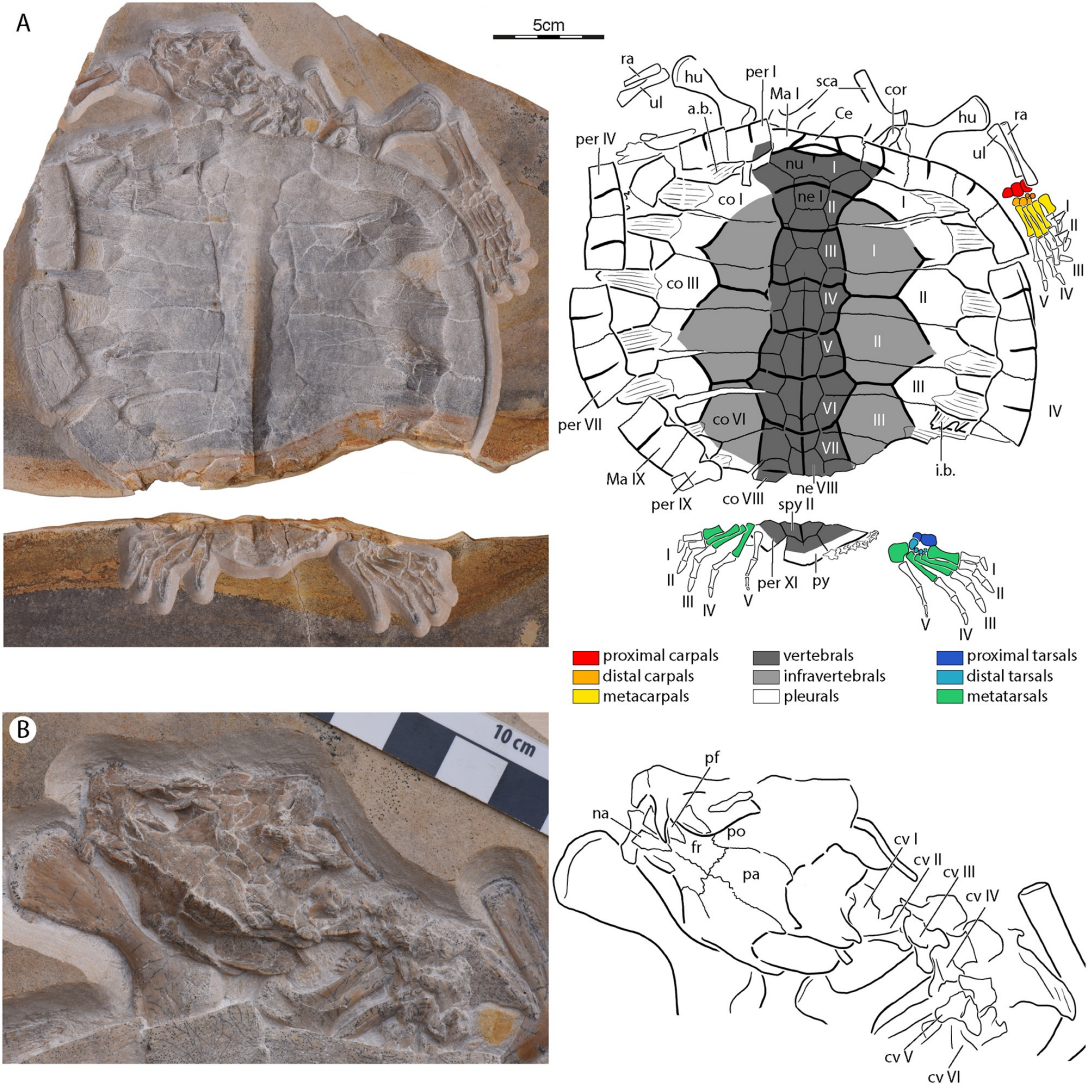
NKMB Watt09/162, *Tropidemys seebachi*, late Kimmeridgian of Wattendorf, Germany. Photograph and interpretive line drawing of (A) complete specimen and (B) skull and cervical column in dorsal view. Abbreviations: a.b., axillary buttress; Ce, cervical scute; co, costal; cor, coracoid; cv, cervical vertebra; fr, frontal; hu, humerus; i.b., inguinal buttress; Ma, marginal scute; na, nasal; ne, neural; nu, nuchal; pa, parietal; per, peripheral; pf, prefrontal; po, postorbital; py, pygal; ra, radius; sca, scapula; spy, suprapygal; ul, ulna. Roman numerals label carapacial scutes and digits.

**Type specimen.** GZG 769–1 (lectotype), anterior portions of a carapace [5, pl. 15.1, 17, pls. 1.1–3, 2.1, 2), designated by [17].

**Type locality and horizon.** Lindener Berg, Hannover, Lower Saxony, Germany; "middle" Kimmeridgian, Late Jurassic [17].

**Distribution.** Late Jurassic (late Kimmeridgian) of Hannover, Lower Saxony (holotype) and Wattendorf, Bavaria, Germany (specimen referred herein).

**Diagnosis.** *Tropidemys seebachi* can most easily be differentiated from all other known representatives of Thalassochelydia by the following combination of characteristics: a small, distinctly keeled, tear-drop shaped carapace (carapace length less than 30cm), thick free rib ends associated with reduced fontanelles, and a complex pattern of carapacial scutes consisting of all least eight vertebrals, three pairs of infravertebrals, four pairs of pleurals, and two serial cervicals.

**Comments.** See Description below for the morphology of the new specimens from Wattendorf and Discussion for the homology of carapacial scute and biogeographic considerations.

## Descriptions

### Achelonia formosa

NKMB Watt15/1 is a partial skeleton consisting of a near-complete carapace, most of the plastron, some remains of the cervical column, a sacral rib, a right hemipelvis, and small, unidentified fragments (Fig 2). The specimen came to rest bottom down and is now embedded in a limestone slab exposed in dorsal view. The dorsal aspects of the carapace are therefore easily observed, but much of the plastron is hidden from view. Assuming that the posterior margin of the carapace was rounded, the specimen can be estimated to have had a median carapace length of 61.5 cm.

Although the posterior margin is missing, the carapace likely once consisted of a nuchal, eleven pairs of peripherals, eight neurals, at least two suprapygal elements, including the "intermediate element," and a pygal (Fig 2). The shell has a circular outline. The nuchal is a rectangular element about twice as wide as long. A minor anterior concavity hints as a small nuchal notch that is formed by the nuchal only. A deep, posterior concavity serves as the articulation site for neural I. The nuchal laterally contacts peripheral I, posteriorly contacts neural I and the medial third of costal I, and contributes on its left side to a carapacial fontanelle.

The neural column is fully preserved in NKMB Watt15/1 (Fig 2). It consists of eight neurals that form an uninterrupted series that fully hinders a midline contact of the costal series. As a general trend, the neurals decrease in midline length, but increase in width from anterior to posterior. The right anterolateral sutures of neural I are not clear, but the remaining sutures suggest that it is a squarish element with an anteriorly convex suture that protrudes deeply into the nuchal, posteriorly converging lateral sutures with costal I, and a posterior convex suture with neural II. While neural II–VI are elongate, hexagonal elements with short anterolateral contacts with the costals, neurals VII and VIII are hexagonal element with similarly sized anterolateral and posterolateral contacts. Neural VIII has a broad posterior contact with the intermediate element.

The costal series is fully preserved on the right side of the specimen, but the distal portions of costals V–VIII are damaged on the left side (Fig 2). The dorsal ribs that form the costals are only partially covered by metaplastic bone. As a general trend, the amount of metaplastic bone decreases from anterior to posterior, with metaplastic bone only covering the medial half in costal I, but only the medial fifth in costal VIII. Large carapacial fontanelles therefore remain open around all costal ribs, include a slit-like, anterior fontanelle formed by peripherals I–III and costal I, and a likely rounded posterior fontanelle formed by the pygal series, peripherals X–XI, and costal VIII. As in the vast majority of turtles, the ribs of costals I–XIII insert distally into peripherals III–X. A general trend is apparent in that the anterior ribs insert more deeply into the peripherals than the posterior ribs. The distal tips of the posterior ribs are therefore visible in dorsal view. The ribs are decorated by pronounced striations. They also appear to be flat, but it is unclear if this is the result of crushing during early diagenesis.

The remains of the pygal series only consist of two elements (Fig 2), which we term the "intermediate element" and "suprapygal I" by reference to other thalassochelydians [19]. The large gap posterior to suprapygal I either suggests the former presence of a large pygal, or the presence of a suprapygal II, in addition to the pygal. The intermediate element is similar in size and proportions to neural VIII. It contacts neural XIII anteriorly, costal VIII anterolaterally, suprapygal I posteriorly, and contributes to the most posterior carapacial fontanelle. Suprapygal I has about the same midline length as the intermediate element, but is much narrower. It has a contact with the intermediate element anteriorly and apparently had a posterior contact with the remainder of the pygal series posterior.

The anterior seven peripherals are preserved on the left side and the anterior ten peripherals on the right side, but the large gap at the posterior margin of the specimen suggests the former presence of an eleventh pair. Most peripherals are preserved in their original anatomical position and therefore only document their dorsal side, with exception of left peripheral IV, which rotated post mortem to expose the lateral side. A minor lip is developed on the left peripheral V and VI, but is absent on the right side. We suggest that this is a taphonomic artifact. The contact with the costal ribs are described above and those with the plastron below. All peripheral contribute to carapacial fontanelles.

Only a few carapacial scute sulci are apparent (Fig 2). A short sulcus can be traced on the nuchal that separated at least one cervical from the first vertebral. The sulcus between vertebral I and II is located on neural I and the most medial portions of costal I and shows a broad, anteriorly directed median inflection. The sulcus between vertebral II and III is the best-developed sulcus and is located on vertebral III and about half of the width of costal III. It lacks a median inflection. A small portion of the sulcus between vertebral III and IV is preserved as well. It is mostly located on neural VI and costal V, but a well-developed, anteriorly-directed inflection crosses onto neural V. Left peripheral IV, the only peripheral that is preserved tipped over on its side, preserves a portion of the sulcus between marginal IV and V. Although no sulci are present that would document the lateral margins of vertebral II and III, a change in surface texture that distinguishes the central from the lateral portions of the carapace highlights areas reminiscent of relatively broad vertebrals, but this may be a preservational artifact.

Portions of both hyoplastra and both hypoplastra can be observed through the carapacial fontanelles (Fig 2). The hyo- and hypoplastra are poorly ossified elements, as the observable axillary and inguinal buttresses consist of a series of finger-like processes that radiate laterally. A large lateral plastral fontanelle therefore appears to be present. Assuming that the hyo- and hypoplastral are preserved in place, the well-developed most anterior process of the hyoplastron articulated bluntly with peripheral II and the poorly developed most posterior process of the hypoplastron with peripheral VII. A small bone is apparent on the posterior left portion of the specimen that we interpret as a xiphiplastron, but a lack of meaningful comparative material makes it difficult to orient this fragment correctly.

At least two cervical vertebrae are preserved along the anterior margin of the specimen, but poor preservation precludes identifying their placement in the column or making meaningful anatomical observations (Fig 2). An isolated sacral rib is preserved at the back of the specimen. In addition, this region documents the right pelvis in medial view. The ilium has a relatively short shaft, suggesting that the shell was not particularly highly domed. The dorsal fan is only moderately expanded towards the posterior. The medial process of the pubis is well-developed, but the posteriorly oriented median process is only short. This suggests that the pubes had a broad midline contact and that the thyroid fenestrae were confluent. The lateral process of the pubis, if present, is protruding into the sediment and therefore hidden from view. The ischium is covered by the pygal series.

### Eurysternum wagleri

NKMB Watt08/406 is a partial skeleton consisting of parts of right costals V–VIII, right peripherals IX–XI, left peripheral XI, parts of the pygal series, and the complete pelvis, hinds limbs, and tail (Fig 3). The specimen came to rest on its back and is now embedded in a limestone slab exposed in ventral view. It is difficult to assess how large this specimen was originally, but comparison with BSPG AS I 921, a specimen of *Eurysternum wagleri* with a near-complete shell, suggests that this specimen once had a carapace with a midline length of about 30 cm.

Only the posterior third of the carapace is preserved in ventral view and much is covered by the pelvis and limbs (Fig 3). It is nevertheless possible to infer that the ribs of costal VI and VII inserted between peripherals VIII and IX and between peripherals IX and X, respectively, that carapacial fontanelles are lacking, and that peripherals XI frame a broad and deep pygal notch. The distal tips of the pygal notch are formed by peripherals XI.

The complete pelvis is present in NKMB Watt08/406, but the right and left halves disarticulated, shifted, and then were crushed (Fig 3). The ilium has a short, but stout neck and the dorsal fan is only moderately expanded towards the posterior. The pubis and ischium have broad midline contacts with their counterparts, but the thyroid fenestrae clearly remained confluent. There is no evidence of an ossified or calcified epipubis. While the pubis possesses a short, but stout lateral processes with a blunt end, the ischium has a short, but pointed metischial processes.

A series of vertebrae are preserved towards the posterior of the specimens that are interpreted as a series of 22 amphicoelous caudal vertebrae preserved in ventral view (Fig. 3). There is no evidence of chevra. The tail appears to be elongate, but this is an illusion created by the displacement of the tail towards the posterior. The small size of the caudal vertebrae relative to those of an unpublished specimen of *Eurysternum wagleri* (CM 3409) suggests that NKMB Watt08/406 is a female.

The limbs disarticulated from the acetabula, but otherwise remain mostly intact (Fig 3). The femur has a rounded head and a clearly developed intertrochanteric fossa. It is unclear if the trochanter major, however, is confluent with the femoral head. The tibia and fibula are about the same length, but the tibia decreases in width, while the fibula increases in width distally. The astragalus and calcaneum are fused. The portion of the astragalocalcaneum formed by the astragalus is about three times larger than that of the calcaneum. The distal tarsals increase in size from digit I to IV. The ansula, the fused distal tarsal V and metatarsal V [38], is a large, blocky, hooked element about the same size as the astragalus. The metatarsals decrease in width from digital I to IV, but increase in length. The pedal formula is 2-3-3-3-4 and digital I–IV bear claws. The metatarsals and phalanges are flattened and lack well-developed articular facets, but this may be a taphonomic artifact caused by crushing. A change in surface texture surrounding the feet is suggestive of soft-tissue preservation.

### Tropidemys seebachi

NKMB Watt09/162 is a nearly complete, crushed skeleton that primarily lacks its left manus (Fig 4). The specimen came to rest on its back and was partially eroded by karstic waters resulting in a broad gap that splits the specimen into two. The specimen is now embedded in two limestone slabs prepared from dorsal. The ventral view is therefore blocked from view. The specimen is estimated to have had a middorsal length of 18 to 18.5 cm.

The cranium is badly crushed and only prepared from dorsal (Fig 4). Only a few morphological details can therefore be observed with confidence.

The skull is longer than broad, but the precise shape is obscured by crushing (Fig 4). The relatively broad interorbital space suggests that the orbits were oriented dorsolaterally. The lower temporal emargination, if present, was minor at best, but the upper temporal emargination approximately reached the level of the foramen stapedio-temporale. A pair of disarticulated nasals are present that roof the external nares. These subtriangular elements likely contacted the maxillae laterally, the frontals posteriorly, and the prefrontals posterolaterally, and one another along the midline. The dorsal plate of the prefrontals are apparent on both sides, but only the right element appears to be in place. The dorsal plate on this side contacts the nasals anteromedially and forms a broad, inset posterior contact with the frontal. The descending branch of the prefrontal cannot be identified. The frontals are large elements that form much of the interorbital space. They form anterior processes that appear to contact the nasals anteriorly and therefore hinder a medial contact of the prefrontals. An anterolateral notch receives the dorsal plate of the prefrontal. The frontals otherwise broadly contribute to the orbit margin, contact the postorbitals posterolaterally, the parietals posteriorly, and their counterpart medially. The dorsal plate of the parietals forms the posterior part of the dorsal skull roof and contributes to the upper temporal emargination. It certainly contacts the frontal and postorbital anteriorly, but its posterior contacts are unclear. The postorbital can only be ascertained to contribute to the orbit and to contact the frontal and parietal laterally. The maxilla appears to form the anteroventral portions of the orbit and to form a well-defined ascending process. The outline of all other bones is unclear.

An elongate element that represents the hyoid is found to the right posterior of the skull, but it is not possible to clarify without seeing the full apparatus if this represents cornu brachiale I or II.

The shell overall has a cordiform outline with a rounded anterior and a pointed posterior (Fig 4). An extremely shallow nuchal notch is formed by the nuchal. A median keel is present that originates at neural III and ends at the pygal. Although the specimen is now crushed, it is apparent that the carapace was tectiform in life. The available cross-section of the shell highlights that the keel is created by a dorsal thickening of the neural series. Carapacial fontanelles are present from costal rib I to costal rib VIII. Scute sulci are clearly impressed toward the center of the shell and its periphery, but not near the carapacial fontanelles. The carapace certainly consists of a nuchal, eight neurals, eight pairs of costals, a suprapygal and a pygal. The size of the gap towards the back of the shell suggests that eleven pairs of peripherals were present, of which peripheral X has eroded completely. It is unclear how many suprapygal elements are missing, but comparison with *Tropidemys langii* suggests the absence of one. We therefore identify the posterior suprapygal as suprapygal II.

The nuchal is rectangular in outline, about twice as wide as long, and forms a shallow nuchal notch (Fig 4). It has a straight lateral contact with peripheral I, a straight posterolateral contact with costal I, and a concave posterior contact with neural I.

The eight available neurals form a continuous series of elements that fully separates the costals from one another (Fig 4). All elements have similar dimensions. Neural I only contacts costals I and therefore has a square outline. Neurals II to VIII are hexagonal. In contrast to most turtles, the anterolateral and posterolateral sides have similar dimensions, although the anterolateral ones tends to be slightly smaller. Neural VIII presumably contacted suprapygal I posteriorly. Neurals III to VIII form a continuous, midline keel that only shows minor notching at the location of the intervertebral sulci. The available cross-section highlights at the level of neural VIII and suprapygal II demonstrated that this keel is formed by a dorsal thickening of these dorsal elements. The keel is rounded anterior, but becomes increasingly sharp towards the posterior of the shell.

The eight pairs of costals are well preserved, with the exception of some damage to the lateral aspects of right costals VI–VIII and left costal VIII (Fig 4). As a general trend, the costals reduce in anteroposterior length from anterior to posterior. The costal increase gradually in mediolateral dimensions from costal I to IV and then decrease strongly in this dimension towards costal VIII. Free rib ends are clearly developed on costals I–VII. A free rib end may also have been present on costal VIII, but the relevant portion of the costal is lacking on both sides of the specimen. The free rib ends are notably rounded, despite strong dorsoventral compression, and strongly striated. They furthermore frame clear carapacial fontanelles that increase in size from costal rib I to III and then strongly decrease in size towards costal rib VII. The displacement of the anterior peripheral combined with crushing makes it unclear if a fontanelle was present anterior to costal rib I, but the well-preserved suture between costal I and peripheral I on both sides of the specimen suggests that this fontanelle was small, if present. The intact posterior margin of the left costal VII and the intact lateral margin of left costal VIII furthermore suggest the presence of a small fontanelle between costal rib VII and VIII. All costals medially contact two neurals, with exception of costal VIII, which has a contact with neural VIII only and likely had a contact with suprapygal I as well. As in most turtles, costals ribs I–VII insert distally into peripherals III–IX, although the ribs of costal III–V are shifted posteriorly to contact the neighboring peripheral as well. Embayments on the dorsal sides of the peripherals that increase in depth from anterior to posterior suggest that the dorsal ribs inserted relatively surficially within the peripherals.

Although the peripheral series is incomplete, the gap between the two existing slabs suggests that eleven peripherals were present, of which peripheral X is eroded completely on both sides of the specimen (Fig 4). The peripherals form a continuous ring that is in contact with the nuchal anteriorly and with suprapygal II and the pygal posteriorly. The medial contacts with the costals are described above.

Karstic weathering fully eroded all of suprapygal I (Fig 4). The intact posterolateral margin of left costal VIII and the partially eroded posterior margin of neural VIII, however, suggests that suprapygal I was a broad element that expanded in width from anterior to posterior. The posterior portions of the damaged suprapygal II document that this element at least had a posterolateral contact with peripheral XI and a posterior contact with the pygal. The pygal is a small, rectangular element that records the posterior termination of the dorsal keel.

The most unusual aspect of NKMB Watt09/162 is its unique pattern of carapacial scutes consisting of at least eight central elements, two rows of at least three costal elements, two serial cervicals, and, presumably, twelve pairs of marginals (Fig 4). The central elements combined with the medial row of costal elements form shapes reminiscent of the broad vertebrals of basal turtles. This may suggest that these two rows are homologous with the vertebrals of other turtles. The central series alone, however, is reminiscent of the narrow vertebrals found in *Tropidemys langii* [39], which may suggest that both rows of costal elements are homologous with the pleurals. As the first hypotheses renders outline more typical of other turtles, we here refer to the central series as the vertebrals, the medial series as the infravertebrals, and the lateral series as the pleurals. Following this terminology, vertebral I has a position reminiscent of vertebral I of other turtles, vertebral II–III together with infravertebral I is reminiscent of vertebral II of other turtles, vertebral IV–V together with infravertebral II is reminiscent of vertebral III of other turtles, and vertebral VI–VII together with infravertebral III is reminiscent of vertebral IV of other turtles.

At least eight vertebrals and four pairs of infravertebrals are preserved on the main slab (Fig 4). The vertebrals are similar in anteroposterior length, but vary in their width, in that square elements tend to be narrower, polygonal elements broader. Vertebral I has an anterior contact with the cervicals, an anterolateral contact with the complete posteromedial rim of margin I, a posterolateral contact with pleural I, and a broad posterior contact with vertebral II. Point contacts furthermore exist with marginal II and infravertebral I. While vertebrals II, III, V, and VII are square elements with only a single lateral contact with an infravertebral, vertebrals IV and VI are hexagonal elements with lateral contact with two infravertebrals. The contacts of vertebral VIII are mostly obscured. The intervertebral sulci are located on neural I, II, III, IV, V/VI, VII, and VIII.

At least three pairs of infravertebrals are present (Fig 4). Infravertebral I has a point contacts with vertebral I, an anterolateral contact with pleural I, a posterolateral contact with pleural II, and three medial contacts with vertebrals II–IV. Infravertebral II has an anterior and posterior contact with infravertebrals I and III, two lateral contacts with pleurals II and III, and three medial contacts with vertebrals IV–VI. Infravertebral III at least has an anterolateral contact with pleural III and two medial contacts with vertebrals VI and VII, but the remaining contacts are obscured.

At least three pairs of pleural elements are present, but their outlines are unclear, as sulci are poorly preserved around the carapacial fontanelles (Fig 4). It is therefore only apparent that pleural I contacts vertebral I anteromedially, infravertebrals I posteromedially, marginal II anteriorly, and pleural II posteriorly, that pleural II contacts pleural I and III anteriorly and posteriorly, and infravertebrals I and II medially, and that pleural III contacts pleural II anteriorly, and infravertebrals II and III medially.

Sulci are not particularly well impressed along the anterior margin of the carapace (Fig 4). We nevertheless identify two medial cervical elements. The anterior cervical form the anterior margin of the shell, contacts marginal I laterally, and the posterior cervical posteriorly. The second cervical is a lenticular element that is squeezed between the anterior cervical and vertebral I. There are vague indications that the anterior cervical is perhaps subdivided into additional cervicals, but we do not have confidence in this observation.

Sulci along the margin of the specimen clearly document the presence of nine pairs of marginals, but three additional pairs likely covered the back of the shell as well (Fig 4). All contacts are obscured with the exception of the contacts of marginals I and II described above.

The plastron remained in situ after death and is therefore almost completely covered from view by the carapace (Fig 4). Parts of the hyo- and hypoplastra can nevertheless be gleaned through some costal fontanelles or ruptured onto the surface of the specimen. The overall shape of the plastron cannot be ascertained. The full exposure of the plastron in the carapacial fontanelles formed by right costal III–V, however, suggests that lateral plastral fontanelles are absent. The left axillary buttress can be seen anterior to costal I and perhaps also anterior to the specimen, but these fragments appear to be displaced. A gap between right costals I and II is suggestive, however, for the presence of an axillary buttress that remains in situ after death and ruptured upwards during taphonomic crushing. This suggests contacts of the axillary buttress with the costals, as in *Tropidemys langii* [39]. Fragments of the inguinal buttresses similarly can be observed at the carapacial fontanelle formed by right costal ribs V and VI, but these, too, appear to be displaced. Instead, a broad gap between left costals VI and V suggests the presence of an inguinal buttress that remained in contact with the costals after death, but rose upwards during taphonomic crushing. Such a contact has also been reported for *Tropidemys langii* [39].

Seven heavily crushed and disarticulated cervical vertebrae are preserved in dorsal view that we interpret as cervicals I–VI (Fig 4). A wing-like process posterior to the right side of the skull documents cervical I, the atlas. Cervical II, the axis, is similarly only documented by its posterior half in dorsal view. Cervicals III–VI are tipped to the side, the neural arches separated from the centra, and much morphology obscured by crushing. We nevertheless can observe that cervicals II–III have large, widely splayed pre- and postzygapophyses and low dorsal processes. It is unclear if transverse processes are present. We also cannot ascertain the presence of formed central articulations.

Five caudal vertebrae in dorsal view are present posterior to the carapace (Fig 4). These elements have narrowly spaced, small pre- and postzygapophyses, small transverse processes located at the middle of the centrum, and low, v-shaped dorsal processes. The central articulations cannot be observed and the presence of chevra cannot be clarified. The tail is not sufficiently preserved to clarify the sex of this specimen.

The left pectoral girdle is only represented by the central portions of the left scapula (Fig 4). The right pectoral girdle is disarticulated, but fully preserved, but the distal ends of the scapular process and coracoid are hidden by the shell. The scapula resembles that of other thalassochelydians by having an angle of about 100 degrees between the acromion and scapular process and an elongate neck above the glenoid.

The humeri can almost completely be observed in dorsal view (Fig 4). The head is rounded, but it is unclear if a shoulder is developed, as the proximal portion of the lateral process is covered by the shell. The two processes can nevertheless be ascertained to splay widely. Neither an ectepicondylar groove or canal can be observed.

The ulna and radius are preserved on both sides of the specimen (Fig 4). The radius is located away from the shell and is distinctly longer than the slightly recovered ulna, which is located closer to the shell. Both elements have slightly expanded ends. The forelimb resembles the paddles of extant marine turtles in that the elbow is overextended.

The slightly disarticulated manus is only preserved on the right side of the specimen (Fig 4). The proximal carpus consists of three elements, the outwardly positioned radiale, the centrale ulnare, and the inwardly positioned pisiform. All three elements are rounded, flattened, and overall similar in size, the ulnare being the largest and the pisiform the smallest element. Two rounded elements with a dense surface texture are tentatively identified as the intermediate carpals. Larger, flattened elements in articulation with metacarpals III–V, on the other hand, are interpreted as distal carpals III–V. Distal carpals I and II are either not ossified, displaced, or still embedded in sediment. All five metacarpals are present. Metacarpal I is the broadest and shortest element. Metacarpal II is slightly longer and narrower. Metacarpals III–V are ever longer and narrower than metacarpal II, but have similar dimensions to one another. We establish a digital formula of 2-2-3-3-3. All five digits bear claws. The digits increase in length from I to IV, but digit V is slightly shorter and slimmer than digital IV. The articulations between the digits are poorly formed, suggesting the presence of a flipper. The combined length of digit IV is about 30% greater than that of the radius and about 60% greater than that of the ulna.

The pelvis, femora, tibia, and fibula either remain hidden from view within the shell, or were eroded by karstic dissolution (Fig 4). The distal pes is preserved on the left side of the specimen and the complete pes on the right. The flattened right astragalus and calcaneum are distinct elements, which suggests that NKMB Watt09/162 is not skeletally mature. The calcaneum is about twice the size of the astragalus. All four distal tarsals are present, although distal tarsal I is barely discernable. Distal tarsal I–III are similar in size and rounded. Distal IV is significantly larger. The pes includes five metatarsals. As a trend, these elements increase in length, but decrease in width from I to IV. The ansula is hook-shaped and larger than the combined astragalocalcaneum. The pedal formula is 2-3-3-3-4. The terminal element on digit V is extremely small on both sides. Pedal claws are developed on digital I–IV. As with the manus, phalangeal articulations are poorly developed, suggesting the presence of a flipper.

## Discussion

### Alpha taxonomy

***Achelonia formosa*.** *Achelonia formosa* was first described from the late Kimmeridgian of Cerin, France [1, 11]. The taxon was initially based on material located on two separate slabs, one containing the anterior portions of a shell, parts of a left forelimb, and a partial cranium and the other consisting of two manus [11], but as the association between these slabs is unclear, the slab with the shell and skull was more recently designated as the lectotype [1]. The original description is associated with idealized drawings that yield little character information [11, pl. 17.5]. We therefore here only compare NKMB Watt15/1 to photographs of the lectotype, while awaiting the long-overdue redescription of this material.

The shell of *Achelonia formosa* only includes the left half of the nuchal, left peripherals I–III, and the distal part of the axillary buttress. The most notable feature apparent from this material is the presence of an extremely poorly ossified costal I coupled with the development of large fontanelles anterior and posterior to the first costal rib. The first costal rib is decorated with numerous ridges and inserts into peripheral III. The first carapacial fontanelle is framed anteriorly by peripherals I–III, posteriorly by the rib of costal I, and laterally by the nuchal. We estimate the width of the carapace at the level of the rib of costal I to have been about 18.5 cm. The historic figure of *Achelonia formosa* [11, pl. 17.5] suggests the presence of a sulcus that runs from the nuchal to the first carapacial fontanelle, but photographs suggest the presence of a narrow cervical and narrow marginals instead.

Though fragmentary, the carapace of *Achelonia formosa* broadly compares to that of NKMB Watt15/1, in particular in the development of an elongate costal rib and large carapacial fontanelles. The most notable difference is size, as the lectotype of *Achelonia formosa* is only 40% of the size of NKMB Watt15/1, whose shell is about 49.5 cm wide at the level of the first costal rib. This is coupled with a disproportionally longer free rib end and better developed carapacial fontanelles in the lectotype, the smaller specimen.

The metaplastic ossification of the costals initially precipitates near the midline and expands laterally during growth. With the exception perhaps of the most neotenic taxa, such as the giant protostegid *Archelon ischyros* Wieland, 1896 [40], all turtles therefore pass the developmental stage apparent in the lectotype of *Achelonia formosa*, either prior to hatching, or as juveniles or subadults. A number of specimens from the Late Jurassic of Europe that were historically classified as "*Aplax oberndorferi*" possess first costals with an even poorer level of ossification [11], but these are neonates or small juveniles with a carapace length of less than 10 cm.

The shell of *Achelonia formosa* can be reconstructed to have belonged to an individual with a shell length between 20 cm (if the shell was circular) to 30 cm (if the shell was more elongated). This allows direct comparison with similarly sized individuals for the vast majority of turtles from the Late Jurassic of Europe, in particular *Idiochelys fitzingeri* [37], *Solnhofia parsonsi* [41], *Tropidemys seebachi* (see this contribution), medium-sized individuals of *Eurysternum wagleri* [18], and smaller sized individuals of *Plesiochelys bigleri* [42] and *Plesiochelys etalloni* [19]. In all cases, the free portions of the first costal rib are short and carapacial fontanelles are reduced to absent. Individuals only about 10 cm larger are available for *Craspedochelys jaccardi* [19] and *Tropidemys langii* [39], but these are unlikely adults of *Achelonia formosa* as they lack any trace of anterior plastral fontanelles as well. Among published material, *Achelonia formosa* therefore only compares with NKMB Watt15/ and, by extension, with the holotype of *Enaliochelys chelonia*.

*Enaliochelys chelonia* was initially named and only briefly described based on material from Ely, Cambridgeshire, United Kingdom [15], but details regarding the morphology of this taxon remained a mystery until parts of the holotype were recently figured and described for the first time [21]. Our comparisons are mostly based on this description, but are supplemented by photographs of the relevant material. The figured portions of the holotype include the nuchal, neurals I–VII, the intermediate element (figured as suprapygal I), the medial portions of costals II-VIII [21, Fig 4a and 4b], a scapula [21, Fig 4c and 4d], and a femur [21, Fig 4c and 4d]. Additional, unfigured remains include fragmentary peripherals (CAMSM J29906), the thickened axillary or inguinal margin of a partial hyo- or hypoplastron (CAMSM J29945), a partial inguinal buttress (CAMSM J29949), two coracoids (CAMSM J29919, 20), a radius and ulna (CAMSM J29929, 30), four phalanges (CAMSM J29933–36), an ilium (CAMSM J29922), a sacral vertebra (CAMSM J29928), six partial caudal vertebrae (CAMSM J29923–27, 47), the occipital portion of a cranium (CAMSM J29947), and numerous herein unidentified bone fragments. Previous studies [1, 21] state by reference to the literature that this specimen is early Kimmeridgian, but that we here conclude this to be an error. The vast majority of fossils collected from Ely are believed to have been collected from the Roswell Pits [43]. Although the lower 2m of sediments from these pits can be assigned to the *Mutabilis* zone, the majority of sediments, and the vast majority of fossil finds, are attributable to the *Eudoxus* zone, which is equivalent to the late Kimmeridgian *Pseudomutabilis* zone [43]. Ely is therefore broadly equivalent in age to Cerin, France and Wattendorf, Germany.

NKMB Watt15/1 resembles *Enaliochelys chelonia* mostly apparently by its large size (ca. 60 cm) and the presence of extremely well-developed carapacial fontanelles. The two specimens further resemble one another by having a square nuchal followed by a rectangular neural I that is deeply inserted into the nuchal, hexagonal neurals II–VI with short anterior sides, poorly developed vertebral sulci, anteriorly directed median inflections for intervertebral sulcus I/II and III/IV, but not intervertebral sulcus II/III, and reduced buttresses consisting of multiple, finger-like projections, as least as partially preserved in CAMSM J29949. We therefore here synonymize *Achelonia formosa* with *Enaliochelys chelonia* based on the three specimens discussed herein from the Late Kimmeridgian of Cerin (France), Ely (United Kingdom), and Wattendorf (Germany).

***Eurysternum wagleri*.** *Eurysternum wagleri* is based on a disarticulated skeleton from the Late Jurassic (*Hybonotum* zone, early Tithonian) of Solnhofen, Germany [8]. This turtle is easily diagnosed from all other named Mesozoic turtles based on the presence of a deep pygal notch [1, 18]. At present, specimens are described from the Late Jurassic of Solnhofen [8], including the type material of *Acichelys redenbacheri* Meyer, 1854 [10], and nearby Denkendorf/Zandt [18, 44], which jointly document most aspects pertaining to the carapace and limbs.

NKMB Watt08/406 resembles the known material of *Eurysternum wagleri* in all regards. Although the view of the posterior margin of the shell is partially blocked by the pelvis, it clearly is characterized by a broad and deep pygal notch. In contrast to the holotype, the pygal notch is not framed by supernumerary peripherals, but this appears to be a polymorphism in the species, as other individuals are known that lack these bones [18]. NKMB Watt08/406 furthermore resembles *Eurysternum wagleri* in that the posterior costals insert between the peripherals, not into the peripherals, the plesiomorphic condition. NKMB Watt08/406 finally resembles previously published specimens of *Eurysternum wagleri* in the proportion of the limbs, although we note that the tiny, supernumerary phalange on the fifth pedal digital renders the digital formal of 2-3-3-3-4 for NKMB Watt08/406, instead of the plesiomorphic 2-3-3-3-3 found in other specimens, but we interpret this as interspecific variation by reference to polymorphism found in extant turtles [45]. NKMB Watt08/406 complements the other material by better documenting the morphology of the pelvis. As the morphology of *Eurysternum wagleri* is so unique among Mesozoic turtles, the attribution of NKMB Watt08/406 to this species is uncontroversial. It also implies a range extension for this species from the early Tithonian into the late Kimmeridgian.

***Tropidemys seebachi*.** *Tropidemys seebachi* was initially based, among others, on a partial carapace, now the lectotype, from the late Kimmeridgian of Hannover, Lower Saxony, Germany [5, 17]. Though fragmentary, the lectotype specimen possesses a rectangular neural I, roughly equidimensional, hexagonal neurals II–IV, a midline ridge formed by a thickening of the neural elements, and a highly unusual scute pattern consisting of at least five anterior vertebrals and five "pleurals" arranged in two rows. It is therefore not surprising that the validity of this species has never been questioned.

NKMB Watt09/162 almost fully overlaps in its morphology with that of the lectotype as recently documented [17]. Three minor differences are apparent. First, NKMB Watt09/162 measures about 7.5 cm from the anterior margin of neural I to the posterior margin of neural IV and is therefore about 30 percent smaller than the lectotype, which measures approximately 11 cm for the same dimensions. Second, vertebral II of NKMB Watt09/162 only has a point contact with pleural I and therefore has a rectangular outline, in contrast to the lectotype, which shows a clear contact of vertebral II with pleural I and the resulting hexagonal outline. And third, a short sulcus suggests the presence of a prepleural in the lectotype located between vertebrals I and II, marginal III and infravertebral I. The presence of a prepleural in the lectotype, incidentally, may be interrelated with the presence of a hexagonal vertebral II. Although these differences may be perceived by some to warrant the naming of a new species, we find greater utility in referring NKMB Watt09/162 to *Tropidemys seebachi*, in part as both individuals originate from deposits of similar age (i.e., late Kimmeridgian). The differences in scute pattern are therefore here interpreted as interspecific variation and difference in size as ontogenetic variation. NKMB Watt09/162 significantly complements knowledge regarding the anatomy of *Tropidemys seebachi* and thereby provide even better taxonomic distinction from other species of *Tropidemys*.

### Biogeography

The herein described material from the early late Kimmeridgian of Wattendorf has important biogeographic implications by suggesting further ties among European turtle faunas of the Late Jurassic at the species level (Fig 1). Although such ties had previously been suggested at the generic level (see [1] for recent summary), explicit ties at the species level have previously only been suggested for *Craspedochelys jaccardi* (France, Portugal, and Switzerland; as first suggested by [24, 46]), *Idiochelys fitzingeri* (France and Germany; as first suggested by [37]), *Plesiochelys etalloni* (France, Germany, Switzerland, and United Kingdom; as first suggested by [25, 37, 47]), *Solnhofia parsonsi* (Germany and Switzerland; as first suggested by [48]), *Tropidemys langii* (Switzerland and United Kingdom, as first suggested by [25]), *Thalassemys bruntrutana* (Switzerland and the United Kingdom, as first suggested by [49]), and *Thalassemys hugii* (Switzerland and United Kingdom, as first suggested by [23]). The herein recognized specimens of *Tropidemys seebachi* provides the first faunal link between northern and southern Germany (as tentatively noted already by [24, 30]) and those of *Achelonia formosa*, including the lectotype of *Enaliochelys chelonia*, provide the first faunal links between France, Germany, and the United Kingdom. The range of *Eurysternum wagleri* remains restricted to southern Germany for the moment. Although we do not recommend using turtles for stratigraphic purposes, it is notable that independent information suggests that all localities that yielded remains of *Achelonia formosa* and *Tropidemys seebachi* are late Kimmeridgian in age.

### Taphonomy

The three herein described specimens from Wattendorf display a broad variety of preservational conditions that are typical for the turtles of this locality.

The specimen of *Tropidemys seebachi* appears to have come to rest fully intact on its dorsal side (it was prepared from the side that was stratigraphically oriented to the bottom in the quarry) and only fell apart slightly post mortem, in particular through the disarticulation of the pectoral elements, the distal phalanges of the right manus, and the left margin of the shell (Fig 4). There are no signs of unhealed breaks that might suggest predation, nor scratch marks that might indicate scavenging. The specimen only presents itself as partially incomplete due to karstic weathering and an excavation-induced absence of the left manus. The cause of death is unclear for this individual.

The specimen of *Achelonia formosa* is a near-complete shell with associated, partially disarticulated cervical and pelvic remains. The shell came to rest ventrally (it was prepared from the side that was stratigraphically oriented to the top in the quarry) and disarticulated slightly along the peripheral series. As the fossiliferous layers are excavated systematically at Wattendorf, it is unlikely that the remainders of the specimen are scattered within the nearer vicinity. The broken posterior ribs on the right side of the specimen may be the result of crushing, but those on the damaged posterior left side of the specimen can only be interpreted as predation marks. There are no signs of bite marks or scavenging. Predation as the cause of death nevertheless appears most likely.

The specimen of *Eurysternum wagleri* is the least complete of the three herein described specimens, as it only consists of the posterior portion of an individual that came to rest dorsally. There are no significant signs of disarticulation or scavenging, but the broken anterior margins of the shell are strong indications for predation, even if bite marks are missing. Here, too, predation seems to be the most likely cause of death.

Although comparative numbers are lacking, few specimens have been described from the plattenkalk of the greater Solnhofen area that display such clear evidence for predation as the cause of death. A notable exception is a pelvis with articulated limbs from Eichstätt [50] that greatly resembles the *Eurysternum wagleri* specimen described herein, but also lacks any shell elements that would allow meaningful taxonomic evaluation. However, given that fossils have never been collected systematically in the quarries of the Solnhofen region, the common occurrence of complete skeletons may well be a collection bias.

The damage to the shell of the *Achelonia formosa* specimens described herein demands a large predator that was able to bite off the posterior third of the shell of this relatively large turtles with a carapace length exceeding 60 cm. A number of large-bodied predators are known to have inhabited the late Kimmeridgian seas of Europe, including *Dakosaurus* spp., a metriorhychid crocodylomorphs that could reach nearly 7 m in length [51], *Machimosaurus* spp., a teleosaurid crocodylomorph that reached up to 6.9 m in length [52], or *Pliosaurus* spp., which could exceed 12 m in length [53]. Although none of these show explicit cheloniovorous adaptations, in particular broad skulls with blunt teeth, their sheer size with skulls easily exceeding 1 m in length renders all possible candidates for predation.
